# Development of syngeneic murine cell lines for use in immunocompetent orthotopic lung cancer models

**DOI:** 10.1186/s12935-020-01503-5

**Published:** 2020-08-28

**Authors:** Kyle Nolan, Gregory Verzosa, Tim Cleaver, Darinee Tippimanchai, Lisa N. DePledge, Xiao-Jing Wang, Christian Young, Anh Le, Robert Doebele, Howard Li, Stephen P. Malkoski

**Affiliations:** 1 Division of Pulmonary Sciences and Critical Care Medicine, University of Colorado Denver Anschutz Medical Campus, 12700 E. 19th Avenue, RC2, Room #9112, Mail stop C272, Aurora, CO 80045 USA; 2 Department of Pathology, University of Colorado Denver Anschutz Medical Campus, Aurora, CO USA; 3 Division of Medical Oncology, University of Colorado Denver Anschutz Medical Campus, Aurora, CO USA; 4 Sound Critical Care, Sacred Heart Medical Center, Spokane, WA USA; 5 Division of Pulmonary Disease and Critical Care Medicine, Virginia Commonwealth University, Richmond, VA USA

**Keywords:** Lung cancer, Mouse models, Orthotopic murine lung cancer models

## Abstract

**Background:**

Immunocompetent animal models are required to study tumor-host interactions, immunotherapy, and immunotherapeutic combinations, however the currently available immunocompetent lung cancer models have substantial limitations. While orthotopic models potentially help fill this gap, the utility of these models has been limited by the very small number of murine lung cancer cell lines capable of forming orthotopic tumors in immunocompetent C57BL/6 hosts.

**Methods:**

Primary lung tumors with specific genetic alterations were created in C57BL/6 background mice. These tumors were then passaged through other animals to increase tumorigenicity and select for the ability to grow in a non-self animal. Once tumors demonstrated growth in a non-self host, cell lines were established. Successful cell lines were evaluated for the ability to produce orthotopic lung tumors in immunocompetent hosts.

**Results:**

We produced six murine lung cancer lines capable of orthotopic lung tumor formation in immunocompetent C57BL/6 animals. These lines demonstrate the expected genetic alterations based on their primary tumor genetics.

**Conclusions:**

These novel cell lines will be useful for evaluating tumor-host interactions, the impact of specific oncogenic alterations on the tumor microenvironment, and immunotherapeutic approaches. This method of generating murine lines capable of orthotopic growth can likely be applied to other tumors and will broaden the applicability of pre-clinical testing of immunotherapeutic treatment regimens.

## Background

Although immunotherapy is the biggest treatment advance in metastatic lung cancer in over 30 years most patients do not respond to this approach and a better basic understanding of tumor-immune interactions is required for immunotherapy to reach its full potential [1]. Unfortunately, the immunocompetent animal models required for these studies are extremely limited. While genetically engineered mouse models (GEMMs) produce tumors in an immunocompetent background, many GEMMs generate multifocal tumors of low malignant potential that may not accurately recapitulate the complex tumor-host interactions present during disease progression [2]. In addition, the low mutational burden of GEMM tumors may limit their utility for studying immunotherapy where therapeutic response is partially dependent on tumor neoantigens [3, 4]. That the viral vectors commonly used to initiate tumor formation also transduce resident immune cells further complicates the use of these models [5, 6]. Finally, generating tumors and monitoring therapeutic responses in GEMMs is complicated and costly.

Orthotopic systems where tumor cells are directly injected into the lungs of recipient mice can also be used to model tumor-host interactions. While this better models metastatic disease and allows for significantly shorter studies then GEMMs [7], this approach has been limited by the small number of transplantable murine lung cancer cell lines. To the best of our knowledge, there are only two commercially available C57BL/6 derived murine lung tumor lines capable of forming orthotopic lung tumors in immunocompetent hosts. The Lewis Lung Carcinoma (LLC) line was sub-cloned from a spontaneous lung tumor in 1951 [8, 9] while CMT167 was sub cloned for metastatic potential from the CMT64 line derived from a spontaneous lung tumor in 1976 [10, 11]. More recently, GEMM-derived lines developed in a mixed genetic background have been described [12–14], however the broad utility of these lines is unclear as these lines may have limited tumorigenicity in C57BL/6 mice. An exception is a *Kras*^*G12D*^*.p53*^*−/−*^ line derived in a C57BL/6 background that forms lung tumors in C57BL/6 mice after tail vein injection [15]. The development of lines capable of orthotopic growth specifically in a C57BL/6 host is critical as many genetic tools for manipulating the murine immune system in vivo exist in this background.

In addition, all the above mentioned cell lines harbor activating Kras mutations [12–16] which may limit generalizability to other oncogenic drivers. Although the mechanistic relationships between oncogenic drivers and immunotherapeutic response remains unclear, human tumors with targetable oncogenic drivers appear poorly responsive to programmed death ligand 1 (PD-L1) blockade [17]. Moreover, the best characterized murine lung cancer cell lines (CMT and LLC) have disparate responses to programmed death ligand-1 (PD-L1) blockade [16], suggesting it will be difficult to discern the relationship between oncogenic driver and immunotherapeutic response without substantial additional tools. Herein, we describe a process for developing murine lung cancer cell lines with a variety of genetic alterations that are capable of forming orthotopic lung tumors in C57BL/6 hosts. This approach will facilitate assessment of tumor-host interactions in the context of different genetic drivers. These lines will be useful for testing combinations of chemotherapy, immunotherapy, and radiation therapy in preclinical models.

## Material and methods

### Mouse strains and background

All studies were IACUC approved (protocol B-95517(05)1E). All strains were backcrossed into C57BL/6 mice (JAX Laboratory, Bar Harbor, ME) until a > 95% C57BL/6 genetic background was obtained by SNP analysis (Dartmouse https://dartmouse.org/). Both male and female animals were used as detailed in “Results”and “Discussion”. Mice with the following alleles were used: *Kras*^*LSL-G12D*^ knock-in (JAX #8179) [18], phosphatidylinositol-4,5-bisphosphate 3-kinase catalytic subunit alpha (*Pi3kca*) mutant knock-in (*R26Stop*^*FL*^P110*, JAX#12343) [19], conditional *TP53* deletion (*p53*^*flox*^, JAX#8462) [20], conditional phosphatase and tensin homolog deletion (*Pten*^*flox*^, JAX#6440) [21], conditional Mitogen-Activated Protein Kinase Kinase Kinase 7 deletion (*Map3k7flox*) [22] (kindly provided by Dr. Scott Cramer, University of Colorado Anschutz Medical Campus), conditional *Smad4* deletion (*Smad4*^*flox*^, JAX#17462) [23], conditional transforming growth factor type II receptor deletion (*Tgfbr2*^*flox*^) [24], mTomato/mGFP (mT/mG) tracking allele (ROSA^mTmG^, JAX #7576) [25]. Genotyping was performed as described in the primary references for the specific alleles.

### Primary tumor formation

Adenovirus with Cre recombinase under the control of the cytomegalovirus (CMV) promoter (Ad5-CMV-Cre) or the surfactant protein C (SPC) promoter (Ad5-SPC-Cre) was purchased from the University of Iowa Viral Vector Core (Iowa City, IA). Adenovirus capable of mediating the echinoderm microtubule-associated protein-like 4 (EML4) anaplastic lymphoma kinase (ALK) gene fusion (Ad-EA) [26] was purchased from Viraquest (North Liberty, IA) with the permission of Dr. Andrea Ventura (Memorial Sloan Kettering). Tumor formation was initiated by injecting 2 µl of virus directly into the left lung or by tracheal instillation as previously described [27, 28] and as detailed in the Results. Cre recombinase viruses were used in animals harboring alleles for conditional oncogene knock-in and/or conditional tumor suppressor deletion while Ad-EA was used in C57BL/6 wild type mice. Animals harboring primary tumors were euthanized 11–36 weeks after tumor initiation.

### Tumor passaging

Primary tumors were dissected from surrounding lung tissue then minced with razor blades. An aliquot of the minced homogenate was suspended in Hanks’ Balanced Salt Solution (HBSS, 14170-112, Gibco, Grand Island, NY) supplemented with 1.3 mg/ml Matrigel (#354234, Corning, Oneonta, NY); 40 µl of this suspension was injected into the left lung of a recipient animal as previously described [7] while 400 µl was injected into the right flank of the same recipient. Animals harboring transplanted tumors were monitored until flank tumor size exceeded 1 cm or until animals showed signs suggestive of internal tumor burden (weight loss, hunched posture) or for up to 9 months. At this point, recipient animals were euthanized and tumors collected for passaging as described above and culture as described below. At each passage, lung tumors > 5 mm were passaged separately (i.e., into separate recipient animals) from flank tumors while lung tumors < 5 mm were combined with flank tumors from the same animal and passaged together (i.e., into the same recipient animal). To reduce the probability of rejection, sex matched recipients were used and all tumor recipients were genetically > 90% C57BL/6 by SNP analysis.

### Cell culture

Cell lines were cultured in Dulbecco's Modified Eagle Medium containing 4.5 g/l d-glucose, l-glutamine, and sodium pyruvate (11885–084, Gibco), supplemented with 10% (v/v) fetal bovine serum (16000-044, Gibco) and 100 μg/ml primocin (ANT-PM2, Invivogen, San Diego, CA) at 37 °C in a humidified atmosphere of 5% CO_2_. Tumors were minced with razorblades then cultured in 6-well plates (CC7682-7506, CytoOne). Lung tumors < 5 mm were combined with flank tumors (from the same animal) for culture while lung tumors > 5 mm were cultured independently from flank tumors.

### Assay for orthotopic tumor formation

Once lines were established in vitro, they were mycoplasma tested, treated if positive, and used between passages 5–10 for orthotopic experiments. Cell suspensions in 50% HBSS/50% Matrigel were created then 500,000 cells in 400 µl were injected into the right flank and 250,000 cells in 40 µl were injected into the left lung as previously described [7]. Recipient mice were monitored until flank tumors exceeded 1 cm or until animals showed signs of internal tumor burden (weight loss, hunched posture) or for up to 45 days. If flank tumors developed too quickly to reliably evaluate lung tumor formation, flank and lung tumor formation was assessed in separate animals. At least four animals (two male and two female) were used to determine the tumorigenicity of each cell line. Lines were deemed successful if they formed tumors in at least 75% of recipient animals.

### Cell line validation

Once the ability to form orthotopic tumors was established, lines were assayed for the anticipated genetic rearrangements by PCR of genomic DNA as previously described: *Kras*^*G12D*^ [29], *Smad4* [30], *Tgfbr2* [31], *Map3k7* [22], *Tp53* [20], *Pten* [21], *R26Stop*^*FL*^P110* [32], *Eml4-Alk* [26]. PCR primer sequences are shown in Table 1. Presence or absence of target gene products (or downstream targets) was also evaluated by Western blotting as previously described [33] using the following antibodies: KRAS^G12D^ (Cell Signaling #14429 1:1000), SMAD4 (Abcam #ab40759 1:5000), TGFBR2 (R&D Systems #AF532), TP53 (Cell Signaling #32532 1:1000), PTEN (Cell Signaling #9559 1:1000), MAP3K7 (Cell Signaling #4505 1:1000), pAKT-Ser473 (Cell Signaling #4058 1:1000), total AKT (Cell Signaling #4691 1:1000), GAPDH (Abcam #ab8245 1:10,000). CMT167 and LLC control cells were kindly provided by Dr. Raphael Nemenoff (University of Colorado Anschutz Medical Campus). These cells were mycoplasma tested upon receipt, treated if positive, and used between passages 10–20 (from receipt).

**Table 1:** PCR primers used to validate cell lines

Target	Recombinant PCR-forward	Recombinant PCR-reverse	Recombinant PCR-reverse 2
p53	CACAAAAACAGGTTAAACCCA	GAAGACAGAAAAGGGGAGGG	
Kras	GGGTAGGTGTTGGGATAGCTG	TCCGAATTCAGTGACTACAGATGTACAGAG	
PTEN	ACTCAAGGCAGGGATGAGC	AATCTAGGGCCTCTTGTGCC	GCTTGATATCGAATTCCTGCAGC
SMAD4	TCCCACATTCCTCTTAGTTTTGA	CCAGCTTCTCTGTCCAGGTAGTA	
PIK3CA	CACAGCTCGCGGTTGAGG	TGCTCGACGTTGTCACTGAA	CGGGTGTACTCCTCATATAACA
TGFβR2	AGGGATGAATGGGCTTGCTT	CTCACCTCAGAGCCTGATTA	
TAK1	GCAACTTCGACAACTTGCCTTCCTGTG	GCACTTGAATTAGCGGCCGCAAGCTTATAACT	
EML-ALK	GAGCCTTGTTGATACATCGTTC	TAGGAGGCAGTTTGGGCTAC	CAAGGCAGTGAGAACCTGAA

To assess GFP expression, cultured cells were heat fixed (95 °C for 5 min) to glass slides, counterstained with DAPI and examined at 510 nm. Western blotting for pAKT and pERK after crizotinib (Selleck, Houston, TX) treatment was performed as previously described [34] using the following antibodies: pAKT S437 (Cell Signaling #4058), total AKT (Cell Signaling #2920), pERK1/2 T202/Y204 (Cell Signaling clone D13.14.4E), total ERK1/2 (Cell Signaling clone L34F12). In vitro cell viability assay was performed as previously described [35]. Briefly, cells were plated into 96-well plates at 1000 cells/well 24 h prior to drug treatment then treated with serial dilutions of the ALK inhibitor TAE-684 (Selleck) for 72 h and viability determined by MTS assay (CellTiter96 AQueous Kit, Promega). Percent inhibition and IC50 were calculated using GraphPad.

## Results

### General approach to development of syngeneic murine lines with orthotopic growth potential

**Fig. 1:**
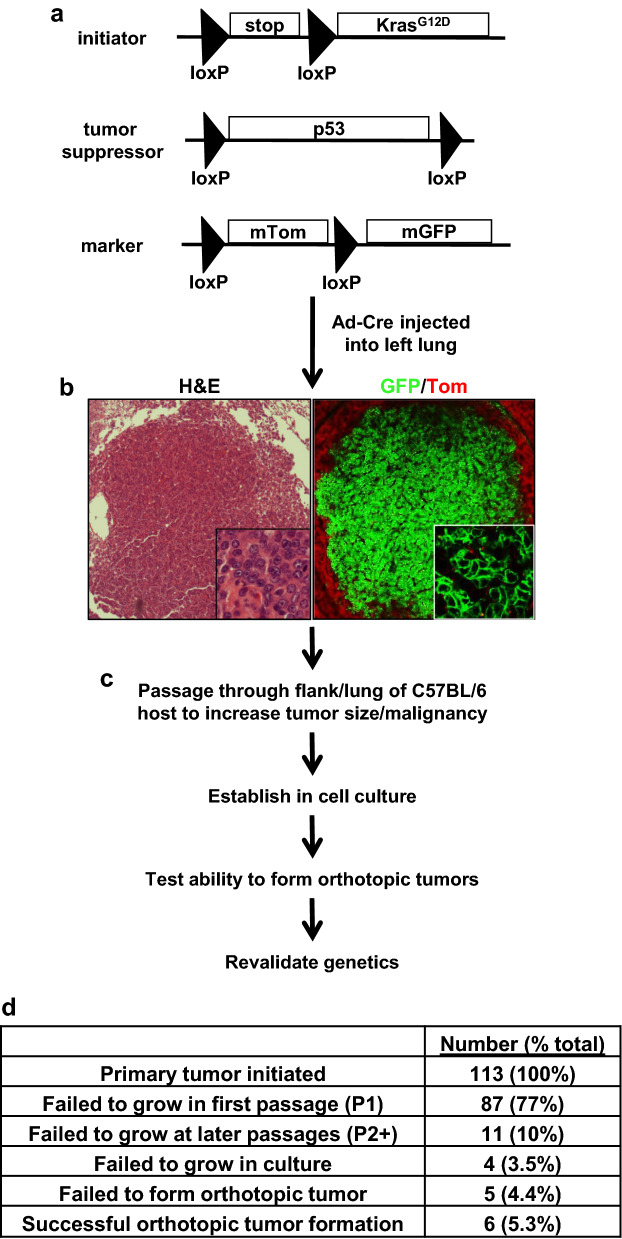
Strategy for developing syngeneic tumor lines with orthotopic growth potential. **a** Example of a genetic background that could be used for primary tumor formation. This animal harbors a Cre-inducible tumor-initiating oncogene (*Kras*^*LSL-G12D*^), a Cre-deletable tumor suppressor (*p53*^*flox*^), and a tracking allele (*ROSA*^*mTmG*^). Homozygous or heterozygous tumor suppressor deletion can promote tumor formation depending on the tumor suppressor. **b** Example of a primary adenocarcinoma showing recombination of the *ROSA*^*mTmG*^ tracking allele. Tumor cells express mGFP while the surrounding non-recombined lung expresses mTomato. **c** Workflow for producing cell lines capable of orthotopic tumor growth. Detailed procedural details are included in the methods. **d** Outcome of attempts to establish cell lines capable of orthotopic tumor formation. There was no discernable pattern or marker that predicted which tumors were likely or unlikely to successfully move through the various stages of development other than establishment of a first passage tumor

We generated mice for primary tumor formation using combinations of conditionally activated tumor-initiating oncogenes (*Kras*^*LSL-G12D*^ or *R26Stop*^*FL*^P110*) and conditionally deleted tumor suppressor alleles (*Smad4*^*flox*^, *Tgfbr2*^*flox*^, *Map3k7flox, PTEN *^*flox*^, *p53*^*flox*^; an example is shown in Fig. 1a). Specific oncogene/tumor suppressor combinations were selected based on prior work [36–38] and ongoing projects. Tumors were initiated by injecting adenovirus that expresses Cre recombinase directly into the left lung [27, 28]. Upon Cre recombinase exposure, oncogenes are activated via excision of an upstream loxP-stop-loxP sequence while tumor suppressors are deleted via LoxP sites surrounding exons. Some animals also harbored a tracking allele (*ROSA*^*mTmG*^) in which cells switch from expressing mTomato to mGFP after recombination; an example of a primary tumor is shown in Fig. 1b.

To enhance the development of tumor lines that could grow in a non-self host, primary tumors were passaged through the flanks and lungs of recipient animals. At each passage after P1, we attempted to establish cell lines from passaged tumors. Once cell lines were established, we tested their ability to form orthotopic tumors. If a cell line was capable of forming orthotopic tumors, we assessed the line for the expected genetic and molecular changes as described in methods and shown in subsequent figures. Workflow is shown in Fig. 1c. Generation of these lines was time intensive, taking 300–500 days from the time that primary tumors were initiated through the time that orthotopic tumor formation was established (Table 2); this excludes time required for breeding and genotyping animals prior to tumor initiation and time for validating lines after orthotopic tumor formation ability was established. This process is also relatively inefficient with only 5% (6/113) primary tumors ultimately leading to lines capable of orthotopic tumor formation (Fig. 1d). Interestingly, the majority of failures (77%; 87/113) occurred at P1; if a successful P1 tumor was established, 6/26 (23%) tumors ultimately led to a cell line capable of orthotopic tumor formation.

**Table 2:** Summary of murine cell lines capable of orthotopic tumor formation

Cell Line	Genotype	Sex	Treatment	Primary tumor size (mm)	Priamary tumor age	P1 time	P2 time	P3 time	P4 time	Total time in vivo	Time in culture	Time in orthotopic	Total time in vitro	Total Time
X577	Kras^G12D^ Smad4 ^*fl/*+^	M	2 ul 10^10^ CMV-Cre	9	184	179	52	35	26	476	23	10	33	509
X911	Kras^G12D^ Tgfbr2^fl/fl^	M	2 ul 10^10^ SPC-Cre	12	202	99	49			350	26	27	53	403
E889	Kras^G12D^ Map3K7fl^/fl^ ROSA^mTmG^	M	2 ul 10^9^ CMV-Cre	3	180	184	54			418	45	45	90	508
X381	Kras^G12D^ Pten ^fl/+^.p53^fl/+^ ROSA^mTmG^	M	2 ul 10^10^ SPC-Cre	1–3	134	135	25	24		318	29	27	56	374
Y856	*R26Stop*^*FL*^P110*^fl/+^ p53^fl/+^	F	2 ul 10^10^ CMV-Cre	6	78	81				159	125	24	149	308
Y143	C57BL/6	F	30ul 5 × 10^6^ Ad-EA	3–5	99	203	46			348	53	25	78	426

All times are in days

### *Development and validation of a Kras*^*G12D*^*.Smad4*^*+/*−^*cell line*

**Fig. 2:**
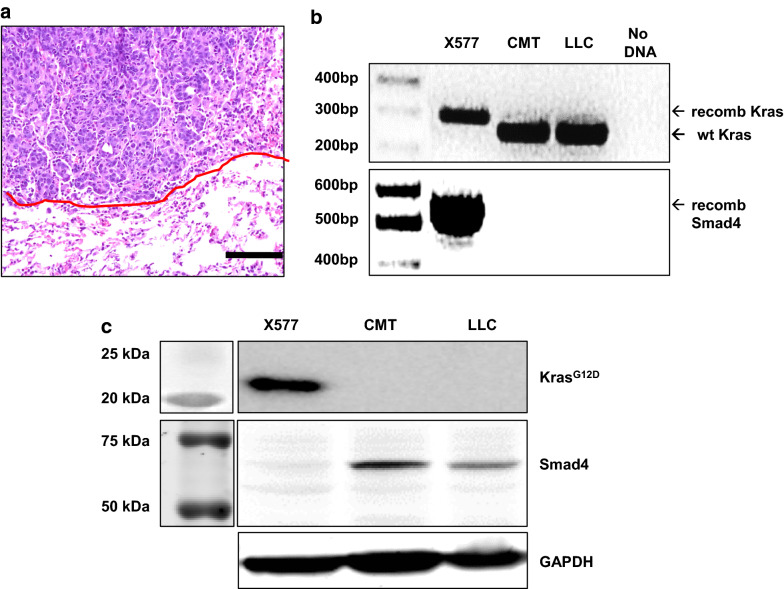
Validation of a *Kras*^*G12D*^*.Smad4*^+/–^ cell line. **a** H&E stain showing orthotopic lung tumor formation by cell line X577. Scale bar is 100 µm and the red line denotes the tumor border. **b** PCR showing the recombinant *Kras*^*G12D*^ allele (305 bp) and *Smad4* allele (500 bp) in X577 cells but not in CMT or LLC controls. Although both CMT and LLC cells harbor *Kras* mutations [16], the 265 bp band in the Kras PCR from CMT and LLC cells represents the wild type (non-engineered) Kras allele. **c** Western blot showing KRAS^G12D^ expression and SMAD4 loss in X577 cells. The KRAS^G12D^-specific antibody detects the KRAS^G12V^ mutation in CMT cells but not the KRAS^G12C^ mutation in LLC cells. The complete absence of SMAD4 expression suggests that the wild type Smad4 allele has undergone mutation, loss of heterozygosity, or transcriptional silencing

A 6 week old *Kras*^*LSL-G12D/*+^*.Smad4*^*fl/*+^ male mouse was injected with 2 µl of 10^10^ PFU/ml Ad5-CMV-Cre into the left lung. When this animal was euthanized 26 week later, a 12 mm primary tumor was passaged into a male recipient animal. After 3 passages through recipient animals (two of which included small lung tumors), cell line X577 was established that was capable of forming orthotopic tumors in immunocompetent C57BL/6 animals (Fig. 2a). As expected, X577 cells demonstrate genetic recombination at the *Kras* and *Smad4* loci (Fig. 2b). By Western blot, X577 cells express KRAS^G12D^ but not SMAD4 (Fig. 2c).

### *Development and validation of a Kras*^*G12D*^*.Tgfbr2*^*−/−*^* cell line*

**Fig. 3:**
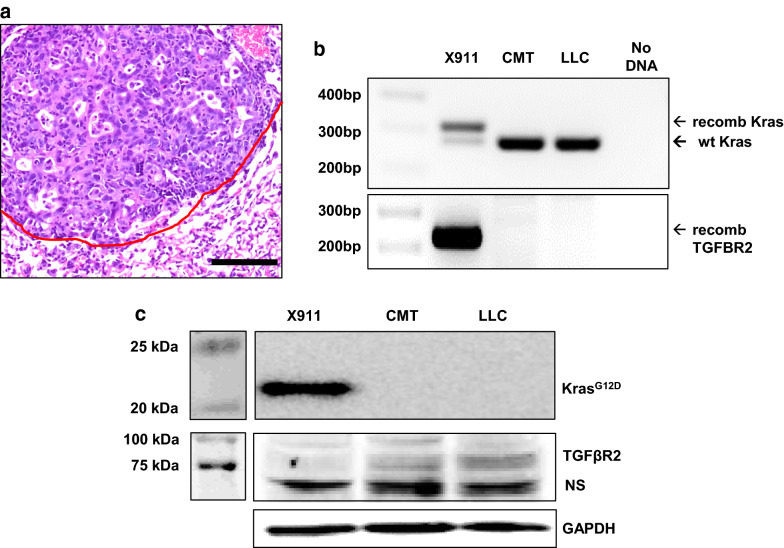
Validation of a *Kras*^*G12D*^*.Tgfbr2*^*−/−*^ cell line. **a** H&E stain showing orthotopic tumor formation by line X911. Scale bar is 100 µm and the red line denotes the tumor border. **b** PCR showing the recombinant *Kras*^*G12D*^ allele (305 bp) and *Tgfbr2* allele (220 bp) in X911 cells but not in CMT or LLC cells. **c** Western blot showing KRAS^G12D^ expression and TGFBR2 loss in X911 cells. The band at ~ 70 kD is nonspecific (NS)

A 6 wk old *Kras*^*LSL-G12D/*+^*.Tgfbr2*^*fl/fl*^ male mouse was injected with 2 µl of 10^10^ PFU/ml Ad5-SPC-Cre into the left lung. When this animal was euthanized 29 week later, a 9 mm primary tumor was passaged into a male recipient. After two passages through recipient animals (one of which included a lung tumor), cell line X911 was established that was capable of forming orthotopic tumors in C57BL/6 animals (Fig. 3a). As expected, X911 cells exhibit genetic recombination at the *Kras* and *Tgfbr2* loci (Fig. 3b) and express KRAS^G12D^ but not TGFBR2 (Fig. 3c).

### *Development and validation of a Kras*^**G12D**^.*Map3k7*^*−/−*^*.GFP*^+^*cell line.*

**Fig. 4:**
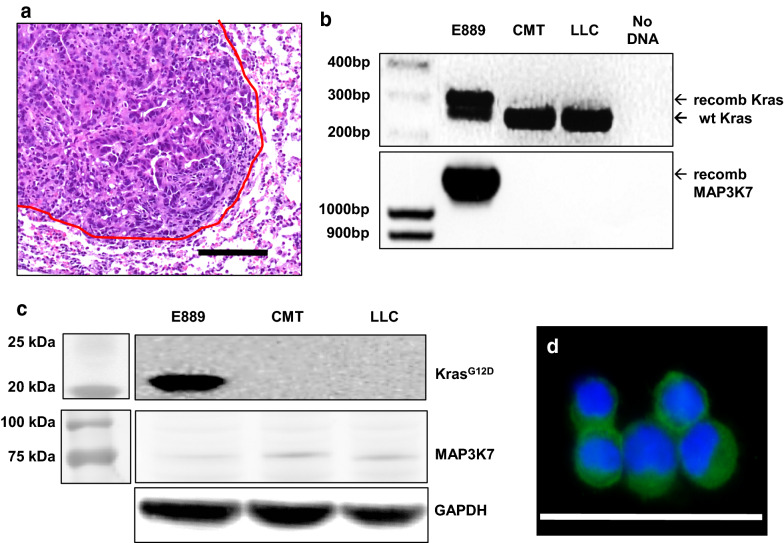
Validation of a *Kras*^*G12D*^*.Map3k7*^*−/−*^.GFP^+^ cell line. **a** H&E stain showing orthotopic tumor formation by line E889. Scale bar is 100 µm and the red line denotes the tumor border. **b** PCR of genomic DNA showing recombination of the *Kras*^*G12D*^ allele (305 bp) and *Map3k7* allele (1.2 kb) in E889 cells but not in CMT or LLC controls. **c** Western blot showing expression of KRAS^G12D^ and loss of MAP3K7 in E889 cells. **d** GFP expression in E899 cells. Cells were heat fixed to a glass slide, counterstained with DAPI, and imaged by fluorescent microscopy; scale bar is 50 µm

An 8 wk old *Kras*^*LSL-G12D/*+^*.Map3k7fl*^*/fl*^*. ROSA*^*mTmG*^ male mouse was injected with 2 µl of 10^9^ PFU/ml Ad5-CMV-Cre into the left lung. When this animal was euthanized 26 week later, an 8 mm primary tumor was passaged into a male recipient. After two passages through the flanks of recipient animals, cell line E889 was established that was capable of forming orthotopic tumors in C57BL/6 animals (Fig. 4a). This line demonstrates genetic recombination at the *Kras* and *Map3k7* loci (Fig. 4b) and expresses KRAS^G12D^ but not MAP3K7 (Fig. 4c) by Western blot. Because this line was derived from an animal harboring the *ROSA*^*mTmG*^ tracking allele [25], it also expresses GFP (Fig. 4d).

### *Development and validation of a Kras*^*G12D*^*.PTEN*^+/−^*.p53*^+/−^*.GFP*^+^*cell line*

**Fig. 5:**
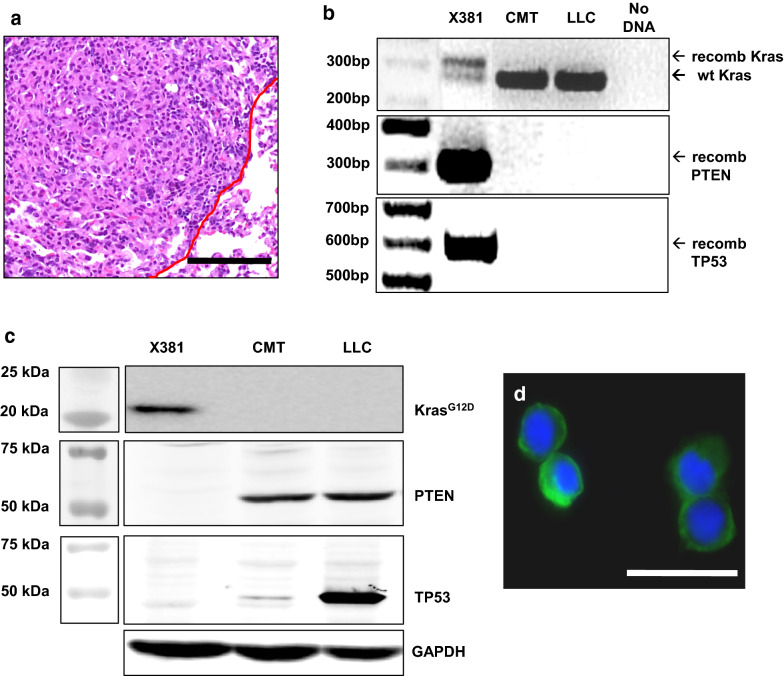
Validation of a *Kras*^*G12D*^*.Pten*^+/−^*.p53*^+/−^.GFP^+^ cell line. **a** H&E stain showing orthotopic tumor formation by line X381. Scale bar is 100 µm and the red line denotes the tumor border. **b** PCR of genomic DNA showing recombination of the *Kras*^*G12D*^ allele (305 bp), *Pten* allele (300 bp), and *Tp53* allele (612 bp) in X381 cells. **c** Western blot showing expression of KRAS^G12D^ and loss of PTEN and TP53 in X381 cells. The absence of detectable PTEN and TP53 expression by Western blot suggests loss of the remaining wild type alleles. Both CMT and LLC cells harbor *Tp53* mutations (Howard Li, personal communication). **d** X381 cells express GFP; scale bar is 50 µm

A 7 week old *Kras*^*LSL-G12D/*+^*.PTEN*^*fl/*+^*.p53*^*fl/*+^*.ROSA*^*mTmG*^ male mouse was injected with 2 µl of 10^10^ PFU/ml Ad5-SPC-Cre into the left lung. When this animal was euthanized 19 week later, multiple primary tumors between 1 and 3 mm were passaged together into a recipient animal. After 3 passages two of which were through the lung, cell line X381 was established that was capable of forming orthotopic tumors in C57BL/6 animals (Fig. 5a). This line demonstrates genetic recombination at the *Kras*, *Pten*, and *Tp53* loci (Fig. 5b). As expected, X381 cells express KRAS^G12D^ but have reduced expression of PTEN and TP53 (Fig. 5c). Because this line was derived from an animal harboring the *ROSA*^*mTmG*^ tracking allele, it also expresses mGFP (Fig. 5d).

### *Development and validation of a Pi3kca*^+^*.p53*^+/−^*cell line*

**Fig. 6:**
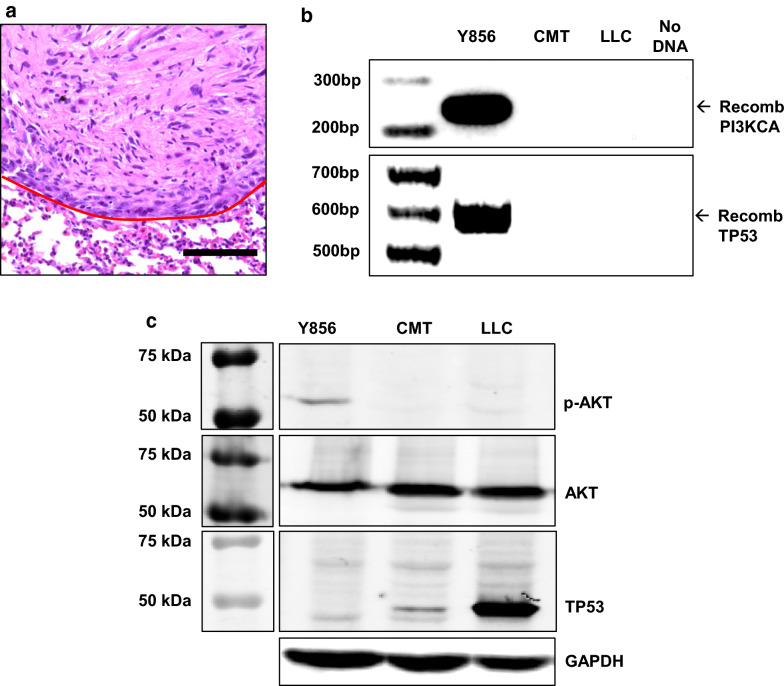
Validation of a *Pik3ca*^+^*.p53*^+/−^ cell line. **a** H&E stain showing orthotopic tumor formation by line Y856. Scale bar is 100 µm and the red line denotes the tumor border. **b** PCR of genomic DNA showing recombination of the *R26Stop*^*FL*^P110* allele (242 bp) and *Tp53* allele (612 bp) in Y856 cells. **c** Western blot showing increased pAKT expression and reduced TP53 expression in Y856 cells

A 20 wk old *R26Stop*^*FL*^P110* ^*fl/*+^*.p53*^*fl/*+^ female mouse was injected with 2 µl of 10^9^ PFU/ml Ad5-CMV-Cre into the left lung. When this animal was euthanized 11 week later, a 6 mm left lung tumor was combined with multiple metastasis (contralateral lung, pericardial, pleural) then passaged into a female recipient animal. After one passage through the flank, cell line Y856 was established; this line was capable of forming orthotopic tumors in C57BL/6 animals (Fig. 6a). Y856 demonstrates genetic recombination of the *Pik3ca* and *Tp53* alleles (Fig. 6b) and reduced TP53 expression (Fig. 6c). Consistent with constitutive PIK3CA activation, Y856 cells demonstrate increase pAKT expression without increased total AKT (Fig. 6c).

### Development and validation of an EML4-ALK mutant cell line

**Fig. 7:**
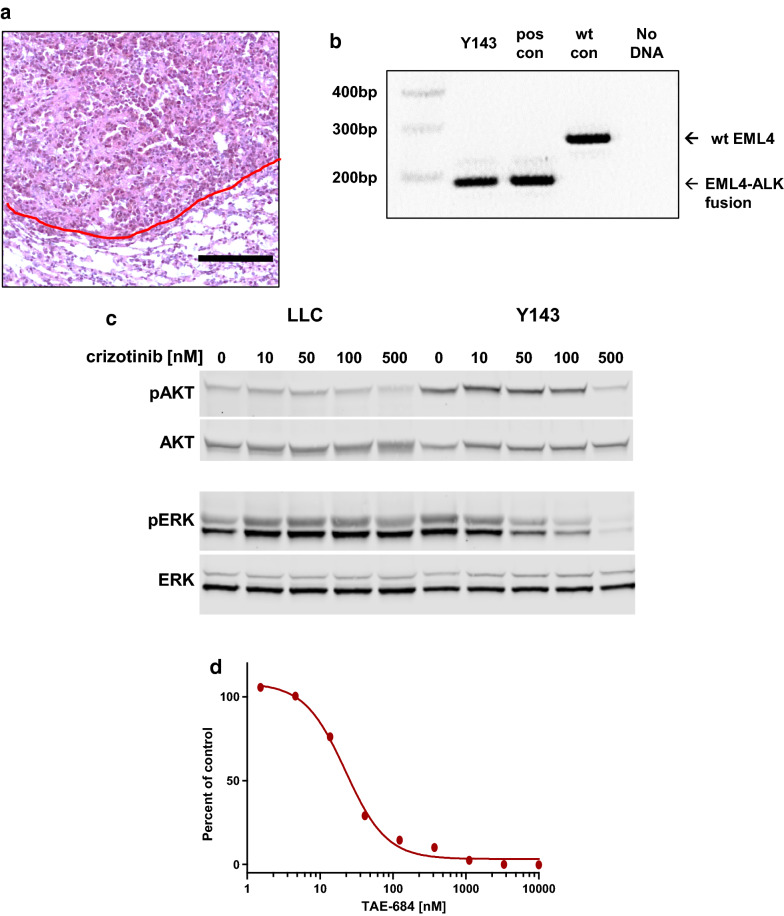
Validation of an EML4-ALK rearranged cell line. **a** H&E stain showing orthotopic tumor formation by line Y143. Scale bar is 100 µm and the red line denotes the tumor border. **b** PCR of genomic DNA demonstrating the 190 bp EML4-ALK rearrangement. Positive control DNA was harvested directly from lung tumors induced by the Ad-EA virus while negative control DNA was harvested from normal mouse lung. **c** Crizotinib treatment reduces pAKT and pERK in Y143 cells. **d** Y143 growth can be inhibited in vitro by TAE684 with an IC50 of 22 nM

An 8 wk old C57BL/6 female mouse was treated with 30 µl of 10^6^ PFU/ml Ad-EA by tracheal instillation as previously described [27, 28]. The Ad-EA vector has an Ad5 backbone and harbors Cas9 and guide RNAs that lead to the EML4-ALK gene fusion [26]. When this animal was euthanized 14 week later a group of multifocal tumors 3–5 mm in size were combined and passaged into a recipient animal. For this line, tumors formed in both lung and flank; these tumors were combined and then passaged together into both lung and flank sites of recipient animals. Subsequently, a cell line (Y143) was established that was capable of forming orthotopic tumors in C57BL/6 animals (Fig. 7a). The EML4-ALK genetic rearrangement was validated using PCR as previously described [26] (Fig. 7b). In Y143 cells treatment with the EML4-ALK inhibitor crizotinib inhibits phosphorylation of AKT and ERK is inhibited in a dose dependent manner (Fig. 7c) and treatment with the ALK kinase inhibitor, TAE684, inhibits growth of Y143 cells (Fig. 7d).

## Discussion

### Rationale for developing syngeneic murine lung cancer cell lines

Better immunocompetent murine lung cancer models are required to study tumor-immune interactions and optimize immunotherapeutic approaches. GEMMs are limited by the production of multifocal tumors of relatively low malignant potential with limited mutational burden [2–4] while orthotopic models are limited by the small number of transplantable murine lung cancer cell lines and limited diversity of driver mutations. Our goal was to develop a systematic approach for producing murine lung cancer cell lines with different genetic alterations that were capable of orthotopic tumor formation in C57BL/6 background recipients. This is of particular relevance as most immune system genetic models exist in a C57BL/6 background and changing the genetic background is labor intensive and expensive. Table 2 summarizes the developmental details of our six novel murine lung cancer lines and illustrates that this process is time intensive, requiring 10–18 months from tumor initiation plus additional time to generate animals for primary tumor formation and validate cell line genetics. Because of the length required to establish successful cell lines, these lines assuredly acquired other genetic alterations that contribute to cell survival, immune evasion, or other characteristics typical of cancers. Transcriptome characterization of lines during development could provide interesting insight into the common pathways required for both in vivo tumor formation and in vitro propagation.

### Developing syngeneic murine lung cancer cell lines: maximizing primary tumor malignancy

Although Kras^LSL-G12D/+^ mice treated with tracheal Ad5-CMV-Cre expire 2–4 months of tumor initiation with lungs that are several times normal in size, most tumors are small adenomas or well differentiated adenocarcinomas [18]. This may explain our limited success in generating tumor lines from Kras^LSL-G12D/+^ animals (not shown). To address this issue, we initiated primary tumor formation via direct injection of virus into the left lung [27]. While tumor production takes significantly longer (4–8 months depending on genotype and virus), this approach allows the development of significantly larger and presumably more malignant tumors. Perhaps not surprisingly, half of our tumor lines originated from primary tumors that were larger than 5 mm (Table 2). That we had two cell lines develop after combining small tumors at the initial passage, suggests this can also be a successful strategy. Although primary tumor histology was not assessed, all orthotopic tumors except Y856 (which may have undergone EMT) had clear adenocarcinoma morphology (see Figs. 2a, 3, 4, 5, 6, 7a). This is consistent the observation that most GEMMs produce predominantly adenocarcinoma spectrum tumors.

### Developing syngeneic murine lung cancer cell lines: minimizing host rejection

We hypothesized that passaging tumors through a second animal would allow for additional tumor growth and might select for tumors more likely to grow in a non-self host, however this approach must consider genetic background as murine lung cancer lines developed from mixed backgrounds [12, 39] do not form orthotopic tumors in C57BL/6 hosts (personal communication Howard Li). Accordingly, we generated primary tumors in animals that were > 95% C57BL/6 by SNP analysis and used tumor recipients for passaging that were > 90% C57BL/6 with no SNP mismatches on chromosome 17 where the mouse major histocompatibility locus (MHC) is located. We also sex-matched primary tumors and tumor recipients to reduce the chances of tumor rejection based on sex specific proteins. Despite these steps, greater than 75% (87/113) of primary tumors failed at the first passage while 23% (6/26) of tumors that successfully completed a first passage ultimately gave rise to lines capable of forming orthotopic tumors. Because our goal was to produce lines capable of orthotopic growth in an immunocompetent host, we did not assess the impact of creating cell lines as the first step in cell line generation.

### Potential utility of syngeneic murine tumors models

The lines described herein are capable of forming both lung and flank tumors in both male and female recipients (not explicitly shown) however there are clear differences in the flank and lung tumor microenvironment and these differences can critically alter immunotherapeutic responses [16]. For monitoring purposes we passaged through flank to allow for tumor amplification and ease of monitoring. While it is unknown what effects this may have had on cell line phenotype, 4 of the 6 lines that were successfully established were also passaged through the lung during development. While all tumor lines formed tumors in at least 75% of recipient C57BL/6 host lungs (not shown); tumorigenicity in other background strains was not assessed. Our goal was to establish tumorigenicity of these cell lines in the lungs of C57BL/6 hosts; future investigators will have to optimize experimental conditions with respect to the number of cells injected and the timing of experimental endpoints that balance primary tumor formation and metastases development.

Although we did not directly compare the responses of the novel cell lines to immunotherapy we did find that IFNγ treatment increases PD-L1 mRNA expression in CMT167, Y856, X577, E889, and X381 cells (Additional file 1: Figure S1); this characteristic is associated with sensitivity to anti-PD-1 treatment in vivo [40]. That LLC and X911 cells fail to respond to INFγ stimulation (and that Y143 cells are equivocal) illustrates how having a broader array of cell lines to test in vivo potentially increases the generalizability of a given observation and also allows investigators to explore mechanistic differences underpinning a specific characteristic. In addition, as these lines have defined oncogenic drivers and retain responsiveness to inhibition of these drivers (at least in the case of the EML4-ALK line), this sets the stage for experiments combining small molecule inhibitor and immunotherapeutic approaches which had previosly been beyond the scope of a typical orthotopic experiment.

## Conclusions

We produced six novel murine lung cancer cell lines capable of orthotopic tumor formation in syngeneic immunocompetent animals. These lines will be invaluable for preclinical studies of small molecule inhibitor and immunotherapy combinatorial approaches. Our methods provide a broader road map for the development of additional murine cancer cell lines capable of orthotopic tumor formation in immunocompetent hosts.

## Supplementary information

**Additional file 1: Figure S1.** Upregulation of PD-L1 in murine lung cancer cells in response to treatment with IFNγ. Cancer cell lines were treated with recombinant murine IFNγ (100 ng/mL R&D Systems) or vehicle for 16 h. RNA was isolated using an RNeasy Mini Kit (Qiagen) and cDNA synthesized using an iScript cDNA Synthesis Kit (Bio-Rad). Real-time PCR analysis was conducted in an iCycler (Bio-Rad). PD-L1 mRNA expression was determined by qRT-PCR and normalized to β-actin. Data represent the mean ± SEM of three independent experiments. Primer sequences: PD-L1 (For: 5′-TGCTGCATAATCAGCTACGG-3′, Rev: 5′-GCTGGTCACATTGAGAAGCA-3′), β-actin (For: 5′-GGCTGTATTCCCCTCCATCG-3′, Rev: 5′-CCAGTTGGTAACAATGCCATGT-3′).

## Data Availability

Data sharing is not applicable to this article as no datasets were generated or analyzed during the current study. Cell lines generated in this study are available from the corresponding author on reasonable request.

## Abbreviations

GEMMs: Genetically engineered mouse models
LLC: Lewis lung carcinoma
PD-L1: Programmed death ligand 1
CMV: Cytomegalovirus
SPC: Surfactant protein C
