# Supplementary figures and images for: Development of syngeneic murine cell lines for use in immunocompetent orthotopic lung cancer models
Source: Cancer Cell Int. 2020 Aug 28;20:417. doi: 10.1186/s12935-020-01503-5 (PMC7455907; doi:10.1186/s12935-020-01503-5)

## Slide 1
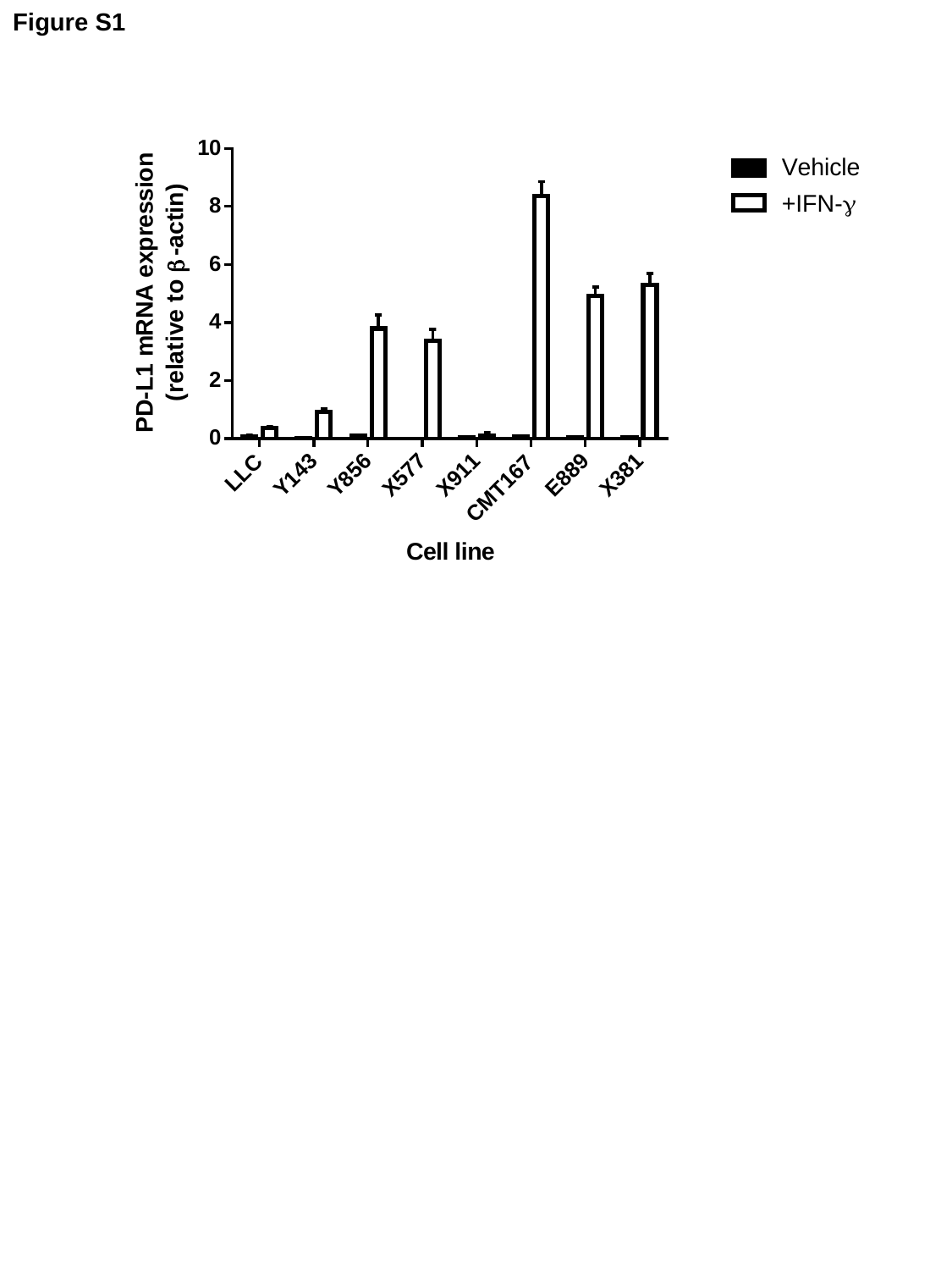

Figure S1

Supplement: Supplementary file 1 — Additional file 1: Figure S1. Upregulation of PD-L1 in murine lung cancer cells in response to treatment with IFNγ. Cancer cell lines were treated with recombinant murine IFNγ (100 ng/mL R&D Systems) or vehicle for 16 h. RNA was isolated using an RNeasy Mini Kit (Qiagen) and cDNA synthesized using an iScript cDNA Synthesis Kit (Bio-Rad). Real-time PCR analysis was conducted in an iCycler (Bio-Rad). PD-L1 mRNA expression was determined by qRT-PCR and normalized to β-actin. Data represent the mean ± SEM of three independent experiments. Primer sequences: PD-L1 (For: 5′-TGCTGCATAATCAGCTACGG-3′, Rev: 5′-GCTGGTCACATTGAGAAGCA-3′), β-actin (For: 5′-GGCTGTATTCCCCTCCATCG-3′, Rev: 5′-CCAGTTGGTAACAATGCCATGT-3′). [file 12935_2020_1503_MOESM1_ESM.pptx]
